# Phage Administration Protects Against *Staphylococcus aureus*-Induced Mastitis Through Direct Pathogen Lysis and Subsequent Alleviation of Host Inflammatory Responses in a Murine Model

**DOI:** 10.3390/foods15213953

**Published:** 2026-11-03

**Authors:** Minfeng Ding, Shuo Li, Yi Liu, Xiaobo Guo, Weican Zhang, Xingping Chen, Xin Li, Tiande Zou, Jun Chen, Jinming You

**Affiliations:** Jiangxi Province Key Laboratory of Animal Nutrition and Feed, Jiangxi Agricultural University, Nanchang 330045, China; dingminfeng0911@163.com (M.D.); lishuo199x@163.com (S.L.); 18870916447@163.com (Y.L.); guoxiaobo93@126.com (X.G.); weicanzhang@stu.jxau.edu.cn (W.Z.); cxp0315@jxau.edu.cn (X.C.); 21917011@zju.edu.cn (X.L.); tiandezou@jxau.edu.cn (T.Z.)

**Keywords:** phage, antibiotic alternatives, mastitis, *Staphylococcus aureus*, transcriptome sequencing

## Abstract

*Staphylococcus aureus* (*S. aureus*)-induced mastitis poses a severe threat to the global dairy industry and raw milk safety. This study aimed to evaluate the protective effects of lytic phage JXAU-SA-P1, recently isolated in our laboratory, against *S. aureus*-induced mastitis in mice and to elucidate its underlying mechanisms. A total of 42 lactating BALB/c mice (7–10 days postpartum) were allocated into seven groups (*n* = 6) to assess bacterial reduction, tissue pathology, and host immune responses following intramammary *S. aureus* challenge. The results showed that JXAU-SA-P1 significantly reduced *S. aureus* burdens and alleviated mammary histopathological damage, with better efficacy than antibiotic therapy. Safety assessments showed that JXAU-SA-P1 was steadily cleared in the absence of host bacteria without triggering inflammation response. Transcriptomic profiling identified 448 overlapping differentially expressed genes (DEGs) modulated by high-dose phage administration. Gene set enrichment analysis (GSEA) revealed that phage administration suppressed major pro-inflammatory pathways, including the TNF, NF-kappa B, and IL-17 signaling pathways. Core regulatory hub genes, including *IL-1β*, *Ptprc*, *Itgam*, *Fcgr3*, and *Itgb2*, were identified through protein–protein interaction network analysis. Subsequent ELISA confirmed significant reductions in pro-inflammatory cytokines (TNF-α, IL-6, and IL-1β), the neutrophil infiltration marker myeloperoxidase (MPO), and serum immunoglobulins (IgA, IgM, and IgG). In conclusion, phage JXAU-SA-P1 effectively alleviated mastitis through direct pathogen lysis and subsequent alleviation of the host inflammatory response, providing a strong experimental foundation for the development of phage-based products as safe, antibiotic-free biocontrol agents for dairy production.

## 1. Introduction

Bovine milk is a vital dietary source of essential nutrients, high-quality proteins, and bioavailable calcium, and plays an indispensable role in the global food supply chain [1]. However, mastitis remains one of the most detrimental diseases affecting the dairy industry worldwide. It not only causes marked reductions in milk yield, but also severely impairs milk composition, processing quality, and dairy cow productivity, resulting in substantial economic losses [2,3]. The majority of mastitis cases are caused by pathogenic microorganisms, with *Staphylococcus aureus* (*S. aureus*) representing a predominant contagious etiological agent [4]. *S. aureus* is transmitted primarily through contact during milking and enters the mammary parenchyma via the teat canal [5]. Importantly, *S. aureus* secretes a range of potent virulence factors, including hemolysins, enterotoxins, toxic shock syndrome toxin-1 (TSST-1), and leukocidins [6]. The persistence of these heat-stable toxins in raw milk can cause severe foodborne intoxications, posing a direct threat to consumer health.

Historically, chemotherapy based on antibiotics has served as the frontline treatment. However, widespread overuse has accelerated the emergence and spread of antimicrobial resistance (AMR) and contributed to the increase in therapeutic failures [7]. Furthermore, intramammary antibiotic administration can result in antibiotic residue contamination in raw milk [8]. Mandatory milk withdrawal periods and the resulting disposal of bulk milk impose additional financial burdens on dairy producers [9]. From a food-processing perspective, residual antibiotics can inhibit the viability of lactic acid starter cultures during cheese and yoghurt fermentation [10], while potentially disrupting gut microbiota homeostasis and facilitating the horizontal transfer of resistance genes to consumers [11]. Consequently, there is a need to develop alternative antimicrobial strategies.

Bacteriophages (phages) are viruses that specifically infect and efficiently lyse bacteria. In recent years, they have garnered considerable attention as potential antimicrobial agents [12]. Compared to antibiotics, phages possess advantages, including self-replication capability, high specificity, potent lytic efficiency, minimal disruption to normal microbiota, coevolution with bacterial hosts, and widespread distribution [13]. Numerous in vitro studies have confirmed their potent bactericidal activity and ability to remove biofilm in various settings [14,15,16,17]. Moreover, phages have shown promising therapeutic efficacy in vivo, reducing bacterial loads and alleviating inflammatory injury [18,19]. Nevertheless, most current studies have focused primarily on pathogen clearance. The precise mechanisms by which phages modulate host transcriptional homeostasis and dampen the devastating cytokine storm in infected mammary tissues remain poorly resolved.

In this study, a lactating mouse model of mastitis was used to assess the efficacy of phage JXAU-SA-P1 in eliminating *S. aureus* colonization and alleviating mammary tissue pathology. By combining bulk RNA sequencing with local and systemic immunological assays, we elucidated the molecular mechanisms through which JXAU-SA-P1 protects the mammary gland by lysing bacteria and suppressing inflammatory signaling pathways. This study provides a theoretical and experimental basis for the application of phage therapeutics in antibiotic-free dairy production and for improving the safety of dairy products.

## 2. Materials and Methods

### 2.1. Phage, Bacterial Strains and Animals

Phage JXAU-SA-P1, which was previously isolated and characterized in our laboratory [16], was propagated using *Staphylococcus aureus* CICC 21600 (China Industrial Culture Collection Center, Beijing, China) as the host strain. The amplified phage lysate was centrifuged, strictly sterilized by filtration through a 0.22 μm PES membrane to ensure the absence of viable *S. aureus*, and subsequently diluted in sterile SM buffer. Lactating BALB/c female mice (7–10 days postpartum) were housed individually in cages under standard laboratory conditions (temperature: 22 ± 2 °C, humidity: 50 ± 10%) with ad libitum access to food and water. The animals were allowed to adapt to the environment for 5 days prior to the experiment. To standardize the physiological status of the mammary glands, the pups were removed and separated from the mother mice 2 h before the formal experimental infection. All animal experiments were approved by the Animal Care and Use Committee of Jiangxi Agricultural University (Approval No. JXAULL-2025091115).

### 2.2. Mouse Model of Mastitis and Experimental Treatments

A total of 42 lactating female BALB/c mice (7–10 days postpartum) were randomly allocated into 7 groups (*n* = 6 per group): PBS control, phage control, *S. aureus*, *S. aureus* + antibiotic, *S. aureus* + low-dose phage (L-Phage), *S. aureus* + medium-dose phage (M-Phage), and *S. aureus* + high-dose phage (H-Phage). The sample size was chosen based on established murine mastitis models to ensure adequate statistical power while adhering to the 3Rs principles [20,21]. The individual mouse was designated as the experimental unit.

The murine mastitis model was established via intramammary infusion, as previously described with minor modifications [20,22]. Briefly, mice were anesthetized by intraperitoneal injection of a mixture of Zoletil 50 (65 mg/kg body weight) and xylazine hydrochloride (10 mg/kg body weight). After disinfecting the teats of the fourth and fifth pairs of mammary glands with 75% ethanol, 50 μL of bacterial suspension or vehicle control was infused into each duct canal using a 32-gauge blunt needle. Mice in the infection groups were challenged with *S. aureus* at 5 × 10^8^ CFU/mL, whereas those in the PBS and phage control groups received an equal volume of sterile PBS and phage JXAU-SA-P1 at 5 × 10^8^ PFU/mL, respectively. After induction of mastitis, analgesia was deliberately withheld as immunomodulatory effects would have directly affected the primary results of the study. To minimize distress, clinical signs including swelling, heat, pain and abnormal milk secretion were monitored every 4 to 6 h. The human euthanasia endpoint was defined as severe lethargy or moribundness. No unexpected adverse events or spontaneous death occurred during the experimental period and no animals were excluded prior to the planned end of the test.

At 4 h post infection, therapeutic formulations (50 μL per gland) were administered through the same route. The PBS control, phage control, and *S. aureus* groups received sterile PBS; the *S. aureus* + antibiotic group received 0.1% (*w*/*v*) ceftiofur sodium (50 μg/gland), based on adaptations from previous studies [23,24]; and the phage treatment groups received phage JXAU-SA-P1 at escalating titers of 5 × 10^6^ (L-Phage), 5 × 10^7^ (M-Phage), and 5 × 10^8^ PFU/mL (H-Phage). At 24 h post-treatment, the mice were anesthetized and euthanized by cervical dislocation. Blood samples were collected to harvest serum, and mammary gland tissues were aseptically dissected for further analyses.

### 2.3. Quantification of S. aureus Loads and Phage Titers

To evaluate bacterial reduction and phage replication in vivo, mammary gland tissues were homogenized in sterile PBS to prepare 10% (*w*/*v*) homogenates. The homogenates were subsequently serially diluted 10-fold in PBS. For bacterial enumeration, 100 μL of 10-fold dilutions were plated on Baird-Parker agar and incubated at 37 °C for 24–48 h. Plates yielding between 30 and 300 colonies were selected for counting, and the results were recorded as CFU/g of tissue. For phage titration, diluted homogenates were analyzed using the double-layer agar method. Similarly, plates exhibiting 30 to 300 plaques were enumerated following overnight incubation, and phage titers were expressed as PFU/g of tissue.

### 2.4. Histopathological Evaluation and Lesion Scoring

During necropsy, mammary tissues were macroscopically inspected for morphological alterations, including changes in tissue color, induration, and the presence of hemorrhagic foci. For microscopic examination, the excised tissues were fixed in 4% paraformaldehyde, dehydrated, and embedded in paraffin. Tissue blocks were stained with hematoxylin and eosin. The severity of tissue damage and inflammation was evaluated and scored based on previously established criteria [25]. Specifically, the evaluation includes three main features. Hyperemia and edema were scored as 0 (normal), 1 (mild), or 3 (severe). Milk stasis and acinar necrosis were graded as 0 (normal), 1 (milk stasis), 2 (moderate), or 3 (severe). Finally, neutrophil infiltration was scored on a scale of 0 to 5, corresponding to 0–1, 2–5, 6–10, 11–15, 16–20, and >20 neutrophils per acinar or mammary gland, respectively. To prevent observer bias, all histopathological evaluations and scorings were performed by an investigator blinded to the experimental groupings.

### 2.5. Transcriptomic Sequencing of Mammary Tissues

Total RNA of mammary tissues was isolated using TRIzol reagent (Invitrogen, Carlsbad, CA, USA) according to the manufacturer’s instructions. RNA concentration, purity, and integrity were evaluated using a NanoDrop 2000 spectrophotometer (Thermo Fisher Scientific, Waltham, MA, USA) and an Agilent 5300 Bioanalyzer (Agilent Technologies, Santa Clara, CA, USA). Four biological replicates per group were selected for RNA-seq. The specific selection was based on optimal RNA integrity and purity to ensure high-quality library construction. High-quality RNA samples (1 μg) were used for stranded mRNA library construction using the Illumina Stranded mRNA Prep, Ligation kit (San Diego, CA, USA). Libraries were quantified using a Qubit 4.0 fluorometer (Invitrogen, Carlsbad, CA, USA) and sequenced on an Illumina NovaSeq X Plus platform (PE150). Raw reads were processed with fastp for adapter trimming and quality control. Raw reads were filtered to remove adaptor sequences and low-quality reads using fastp. The resulting clean reads were mapped to the *Mus musculus* reference genome (assembly GRCm39) in a directional mode using HISAT2, and transcripts were assembled and quantified using StringTie software (v2.2.3).

### 2.6. Identification of Differentially Expressed Genes (DEGs) and Functional Enrichment

Principal component analysis (PCA) was performed to assess sample clustering and overall variance among biological replicates. Differentially expressed genes (DEGs) among treatment groups were identified using DESeq2, with a threshold of |log_2_FoldChange| > 2 and FDR < 0.05. Shared and uniquely regulated DEGs across groups were identified and depicted using Venn diagrams. GO functional annotation and KEGG pathway enrichment analyses were conducted using Goatools (v1.1.5) and Python (v3.13.1) scipy package, respectively. Gene set enrichment analysis (GSEA) was subsequently performed against the KEGG pathway database, the activation or suppression of specific signaling cascades was determined based on the sign of the normalized enrichment score (NES). Furthermore, a protein–protein interaction (PPI) network was constructed using the STRING database with a high-confidence interaction score threshold (>0.70) and visualized using Cytoscape software (v3.10.5).

### 2.7. RT-qPCR Analysis

To validate the transcriptomic findings, total RNA was extracted from the mammary tissues using TRIzol reagent. Following genomic DNA clearance, total RNA was reverse-transcribed using a commercial reverse transcription kit (Accurate, Changsha, China). RT-qPCR were performed on a QuantStudio™ 5 Real-Time PCR System (Thermo Fisher, Waltham, MA, USA) using the 2× SYBR Green qPCR mix (Accurate, Changsha, China). Primer sequences were designed using the Primer-BLAST online tool (https://www.ncbi.nlm.nih.gov/tools/primer-blast/ accessed on 4 March 2025) and synthesized by Sangon Biotech Co., Ltd. (Shanghai, China). The primer sequences are detailed in Table 1. The relative mRNA expression levels of target genes were normalized to the reference gene *β-actin* and calculated using the 2^−ΔΔCT^ method [24].

### 2.8. Enzyme-Linked Immunosorbent Assay (ELISA)

To evaluate inflammatory responses, mammary tissues were homogenized to generate 10% (*w*/*v*) homogenates. Total protein concentrations in the tissue extracts were determined using a BCA protein assay kit (Beyotime, Shanghai, China). The levels of immunoglobulin A (IgA), immunoglobulin G (IgG), immunoglobulin M (IgM), tumor necrosis factor-alpha (TNF-α), interleukin-1 beta (IL-1β), interleukin-6 (IL-6), and myeloperoxidase (MPO) in the serum, alongside TNF-α, IL-1β, IL-6, and MPO in the mammary tissue homogenates, were quantified using target-specific ELISA kits (Shanghai Enzyme-linked Biotechnology Co., Ltd., Shanghai, China) following the manufacturer’s instructions. Tissue cytokine levels were normalized to the corresponding total protein concentration of the homogenates.

### 2.9. Statistical Analysis

All statistical analyses were conducted using SPSS Statistics software (v27.0). The normality of the data distribution and the homogeneity of variances were confirmed using the Shapiro–Wilk test and Levene’s test, respectively. Statistical differences among multiple groups were evaluated using a one-way analysis of variance (ANOVA), followed by Tukey’s post hoc test. Quantitative data are presented as the mean ± standard error of the mean (SEM). Statistical significance was considered as *p* < 0.05.

## 3. Results

### 3.1. Therapeutic Efficacy of Phage JXAU-SA-P1 in S. aureus-Induced Murine Mastitis

**Figure 1:**
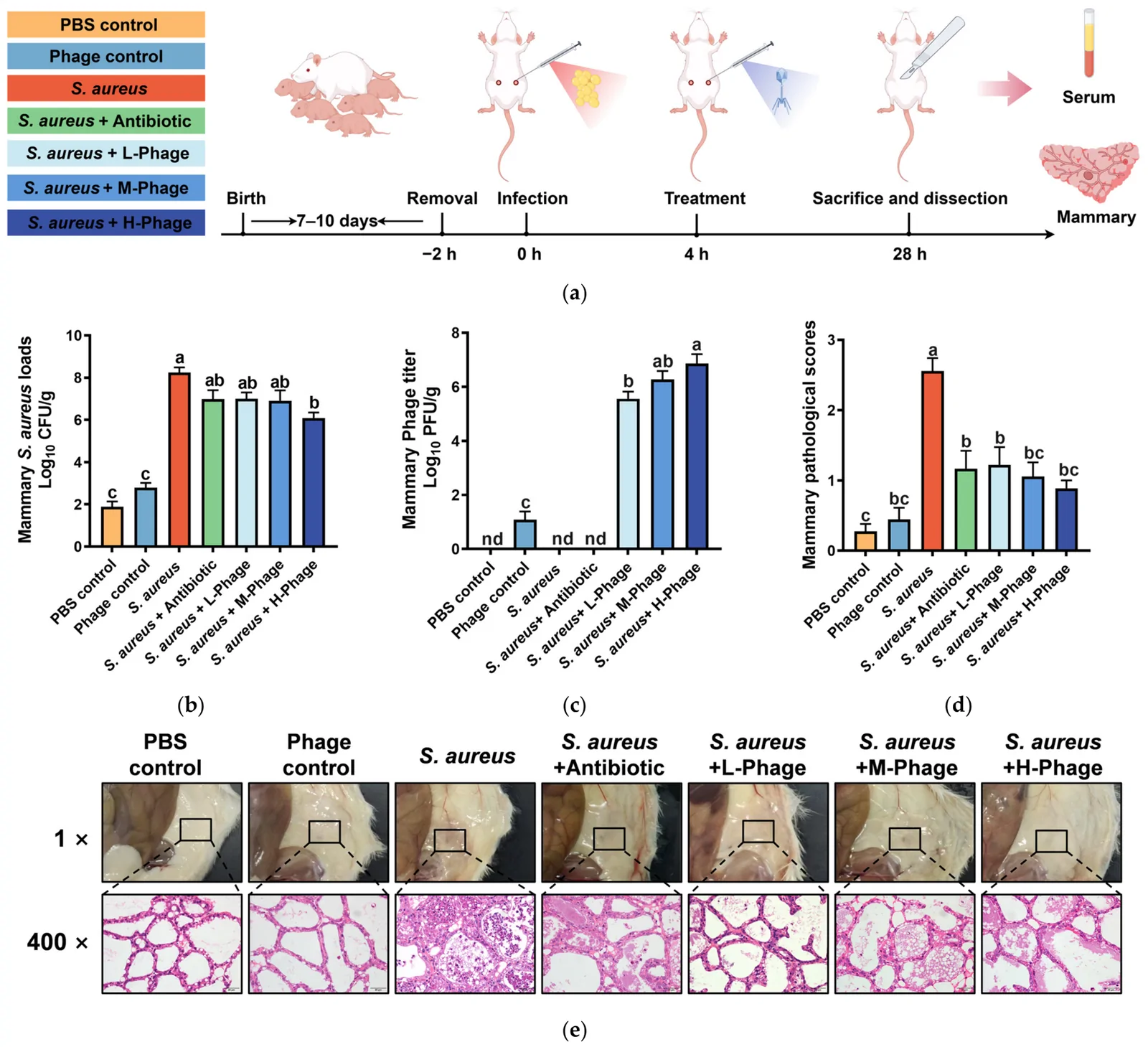
Therapeutic efficacy and tissue-protective effects of phage JXAU-SA-P1 against *S. aureus*-induced mastitis (mean ± SEM; *n* = 6). (**a**) Schematic diagram of evaluating the protective effects of phage JXAU-SA-P1 against *S. aureus*-induced mastitis in mice. (**b**) *S. aureus* loads in mammary tissue. (**c**) Phage titers in mammary tissue. (**d**) Mammary pathological scores. (**e**) Images of mouse mammary glands and H&E staining (scale bar = 20 μm). “nd” stands for “not detected”. Different superscript letters indicate statistical differences among treatment groups (*p* < 0.05).

We assessed the in vivo therapeutic potential of phage JXAU-SA-P1 using a murine model of *S. aureus*-induced mastitis (Figure 1a). While both the antibiotic and varying doses of phage treatments trended toward reducing the bacterial load relative to the untreated infected group, the high-dose phage (H-Phage) intervention yielded the most robust and statistically significant bacterial reduction (*p* < 0.05, Figure 1b). Assessment of local phage titers (Figure 1c) revealed a clear dose-dependent accumulation in the mammary tissues, reaching 5.60, 6.28, and 6.86 log_10_ PFU/g in the L-Phage, M-Phage, and H-Phage groups, respectively. Notably, despite receiving the same dose, the phage titer in the phage control group (0.93 log10 PFU/g) was significantly lower than that in the *S. aureus* + H-Phage group (*p* < 0.05), indicating rapid in vivo phage clearance in the absence of a host.

Macroscopic and histopathological evaluations further confirmed the protective effects of the phage (Figure 1d,e). The PBS and phage control groups exhibited normal, milky-white mammary tissues with intact alveolar structures and no obvious pathological lesions, indicating the high biocompatibility of phage JXAU-SA-P1. Conversely, the *S. aureus* group presented with severe hyperemia, tissue induration, and deep red discoloration. Histological examination of the infected glands revealed catastrophic disruption of the acinar structures, abundant necrotic debris, and massive leukocytic infiltration within the alveolar lumen and interstitial stroma. Therapeutic intervention, particularly with high-dose phage, dramatically reversed this infection-induced tissue damage. The mammary tissues largely regained their normal color with only minor hemorrhagic foci, and histopathological analysis revealed relatively intact acini with significantly reduced leukocytic infiltration. Consequently, the pathological scores in all treatment groups were significantly lower than those in the untreated infection group (*p* < 0.05), with the H-Phage group exhibiting the most pronounced tissue recovery. Collectively, these findings demonstrate that high-dose phage therapy effectively restricts *S. aureus* colonization and mitigates local tissue damage in vivo.

### 3.2. Transcriptomic Analysis of Mammary Tissues

**Figure 2:**
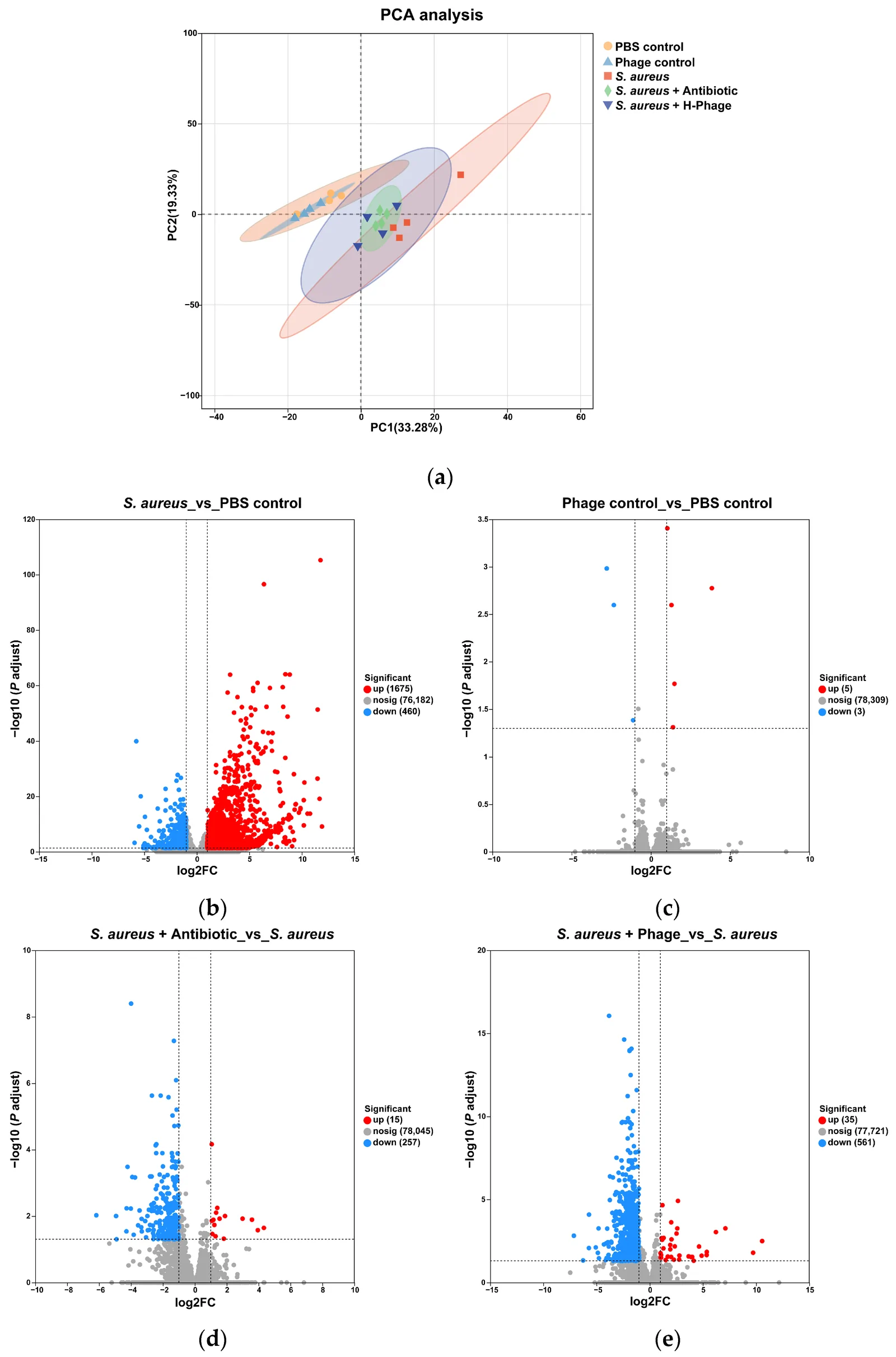
Principal component analysis (PCA) and volcano plot analyses of differentially expressed genes (DEGs) among experimental groups (*n* = 4). (**a**) PCA. (**b**–**e**) Volcano plots of differentially expressed genes for *S. aureus* vs. PBS control, phage control vs. PBS control, *S. aureus* + antibiotic vs. *S. aureus* and *S. aureus* + H-Phage vs. *S. aureus* groups.

To elucidate the underlying mechanisms driving this protective effect of phage JXAU-SA-P1, transcriptomic analysis was performed on mammary tissues from the PBS, phage control, *S. aureus*, *S. aureus* + antibiotic, and *S. aureus* + H-Phage groups. The sequencing yielded 130.96 Gb of high-quality clean data, with an average output of ≥ 5.48 Gb per sample. Across all libraries, the Q20 and Q30 base percentages exceeded 99.32% and 96.62%, respectively (Table 2). Furthermore, 97.40–97.90% of the clean reads mapped uniquely to the *Mus musculus* reference genome (Table 3), confirming the high fidelity and reproducibility of the transcriptomic dataset. Principal component analysis (PCA) revealed distinct transcriptomic signatures across the treatment groups (Figure 2a). Biological replicates within each group clustered tightly, while clear spatial separation was observed between different groups, reflecting the stability of the disease model and profound transcriptional reprogramming induced by the treatments.

Differentially expressed genes (DEGs) were subsequently identified using DESeq2 (|log_2_FoldChange| ≥ 2, adjusted *p* < 0.05). *S. aureus* challenge induced widespread transcriptional perturbation, resulting in 2135 DEGs (1675 up-regulated, 460 down-regulated) compared to the PBS control (Figure 2b). Notably, administration of phage alone exerted negligible impact on the basal transcriptome, with only eight DEGs detected between the phage control and PBS control groups (Figure 2c), strengthening the preliminary evidence of in vivo safety of phage JXAU-SA-P1. Following therapeutic intervention, 272 DEGs (15 up-regulated, 257 down-regulated) were identified in the *S. aureus* + antibiotic group relative to the *S. aureus* group (Figure 2d). Alongside, the *S. aureus* + H-Phage group exhibited 596 DEGs, the vast majority of which were down-regulated (35 up-regulated, 561 down-regulated) (Figure 2e). This pronounced bias toward gene suppression indicates that high-dose phage administration generally reduces the hyperactive expression of infection-driven genes.

### 3.3. Functional Enrichment Analysis Reveals the Suppression of Inflammatory Pathways by Phage Treatment

**Figure 3:**
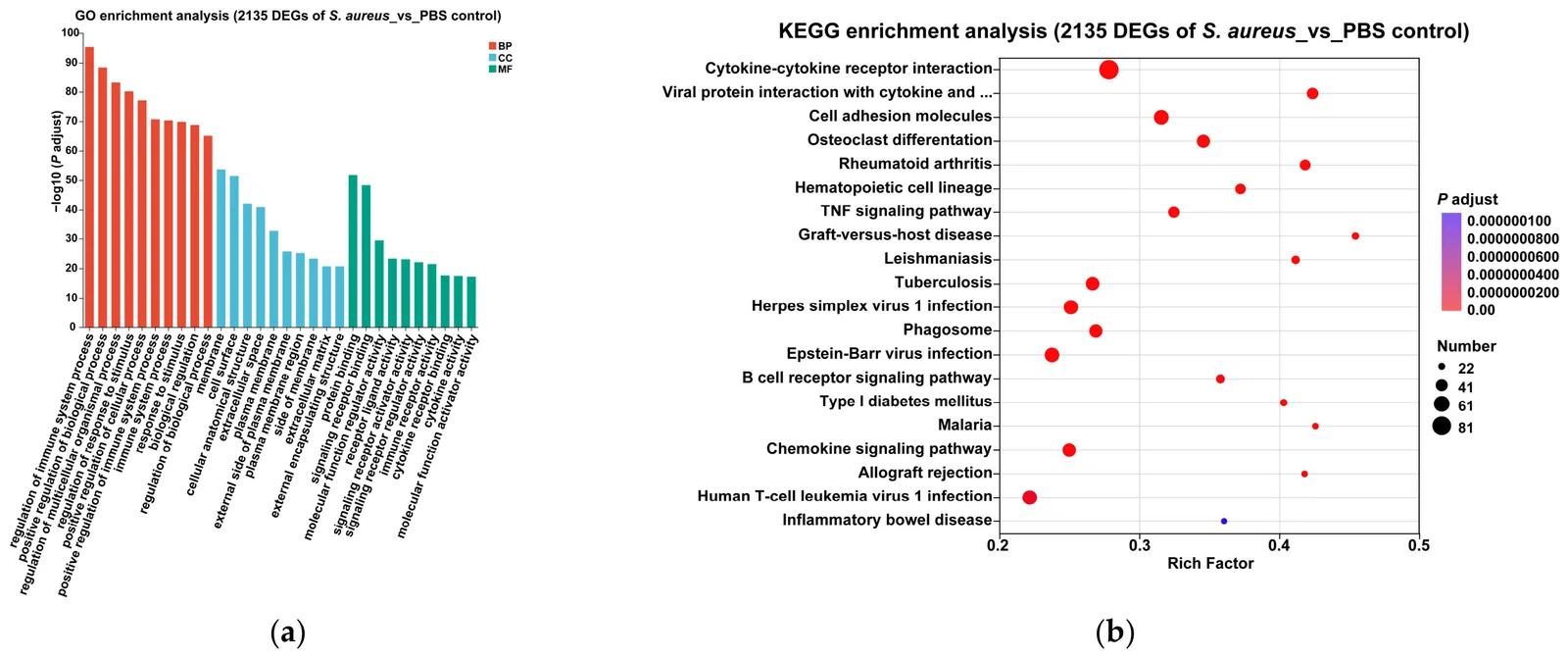
GO functional annotation and KEGG pathway enrichment analysis of DEGs between *S. aureus* and PBS control groups. (**a**) GO functional annotation. (**b**) KEGG pathway enrichment.

GO and KEGG functional enrichment analysis were further performed on the 2135 DEGs between the *S. aureus* and PBS control groups (Figure 3). In the biological process (BP) category, the DEGs were predominantly enriched in immune response regulation, positive regulation of biological processes, and response to external stimuli. Cellular component (CC) terms were mainly associated with the plasma membrane, cell surface, and extracellular space, while molecular function (MF) analysis highlighted significant enrichment in signaling receptor binding, cytokine receptor binding, and cytokine activity. KEGG pathway enrichment analysis revealed that the 2135 DEGs were significantly enriched in multiple signaling cascades that are linked to host innate immunity and inflammatory responses. Notably, these pathways included cytokine–cytokine receptor interaction, the TNF signaling pathway, the chemokine signaling pathway, the JAK-STAT signaling pathway, and the IL-17 signaling pathway. These results highlight that *S. aureus* intramammary challenge triggers robust local immune responses and activates multiple pro-inflammatory signaling pathways.

**Figure 4:**
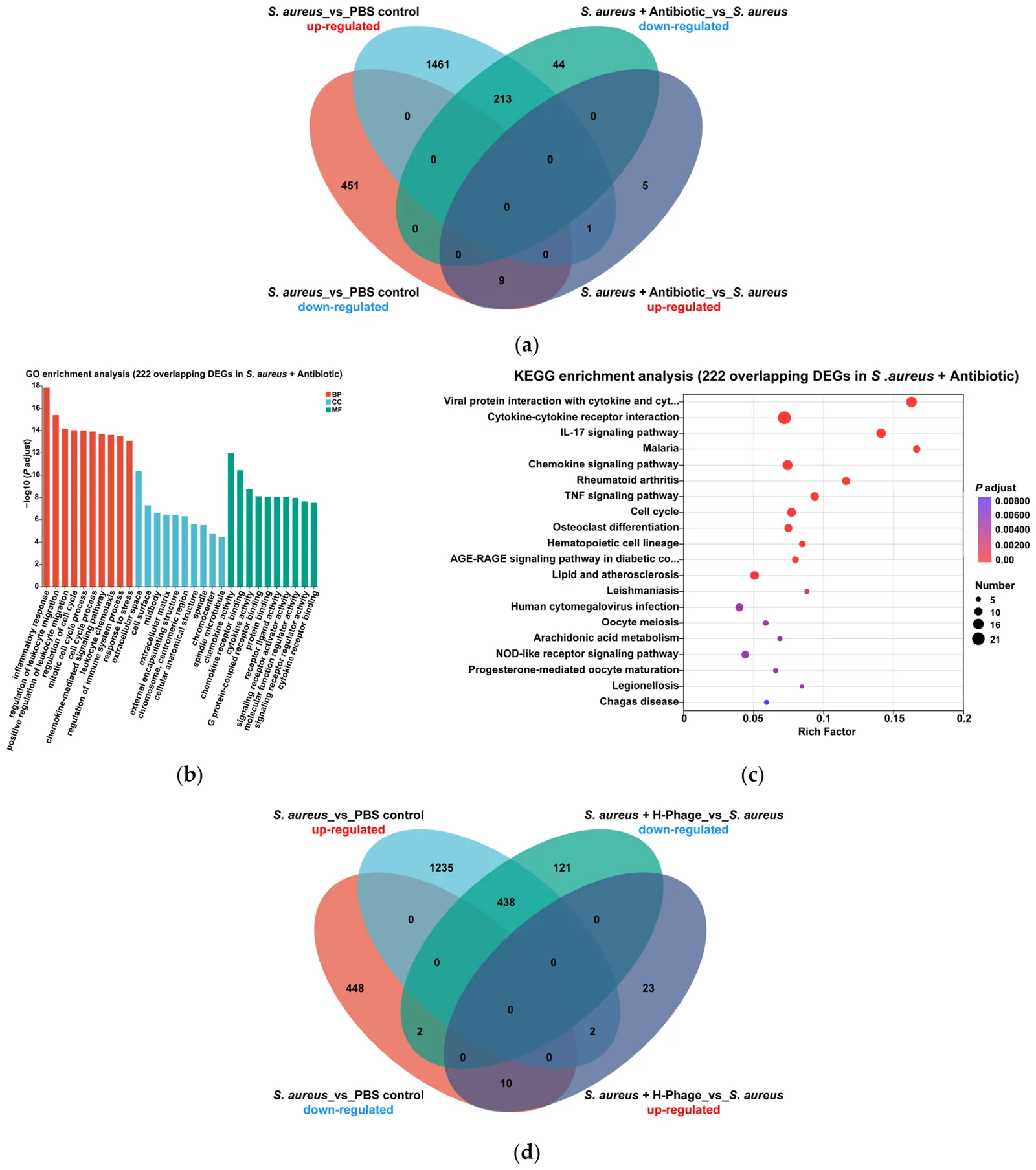

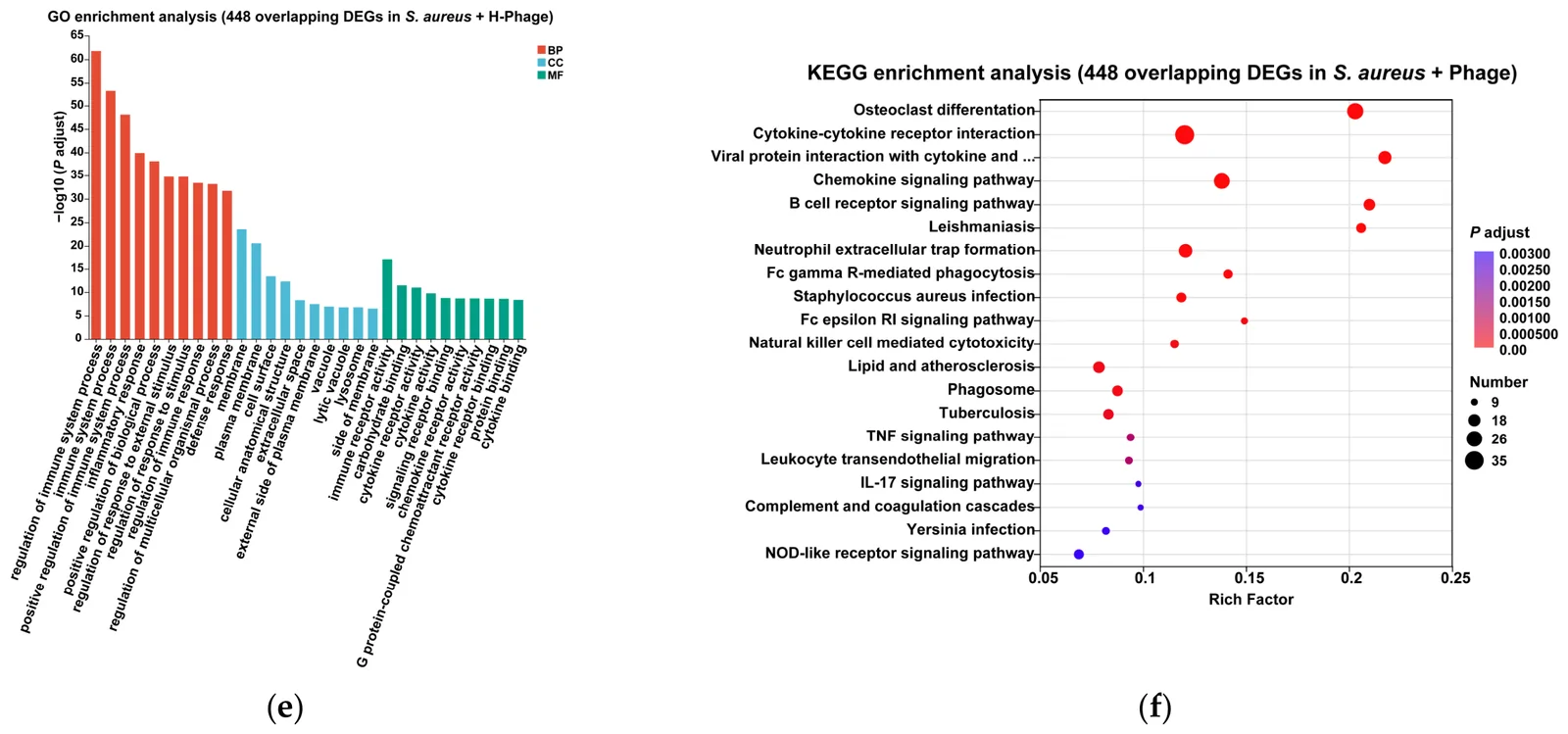
Venn diagrams and functional enrichment analyses (GO and KEGG) of overlapping DEGs under antibiotic and phage therapies. (**a**) Venn diagram of DEGs between *S. aureus* vs. PBS control and *S. aureus* + Antibiotic vs. *S. aureus* group. (**b**,**c**) GO functional annotation and KEGG pathway enrichment analysis of overlapping DEGs under antibiotic treatment. (**d**) Venn diagram of DEGs between *S. aureus* vs. PBS control and *S. aureus* + H-Phage vs. *S. aureus* group. (**e**,**f**) GO functional annotation and KEGG pathway enrichment analysis of overlapping DEGs under high-dose phage treatment.

Venn diagram analysis was conducted to identify the core transcriptional genes reversed by therapeutic interventions. As shown in Figure 4a, antibiotic treatment effectively normalized the expression of 222 infection-induced DEGs (213 up-regulated and 9 down-regulated). GO enrichment analysis revealed that these 222 reversed DEGs were primarily associated with biological processes (BP) such as inflammatory response, regulation of leukocyte migration, and cell cycle regulation; cellular components (CC) including the extracellular space and cell surface; and molecular functions (MF) dominated by chemokine activity, chemokine receptor binding, and cytokine activity (Figure 4b). KEGG enrichment is mapped to cytokine–cytokine receptor interaction, the chemokine signaling pathway, and the TNF signaling pathway (Figure 4c).

Notably, high-dose phage treatment induced an extensive transcriptional reversal, effectively reversing 448 DEGs (438 up-regulated and 10 down-regulated, Figure 4d). GO enrichment demonstrated that these 448 overlapping DEGs were predominantly involved in immune and inflammatory processes, including regulation of immune system process, inflammatory response, and defense response in the BP category, the membrane, plasma membrane, and cell surface in the CC category, and immune receptor activity and cytokine receptor activity in the MF category (Figure 4e). Consistently, KEGG enrichment mapped these genes to cytokine–cytokine receptor interaction, chemokine signaling pathway and *Staphylococcus aureus* infection (Figure 4f).

**Figure 5:**
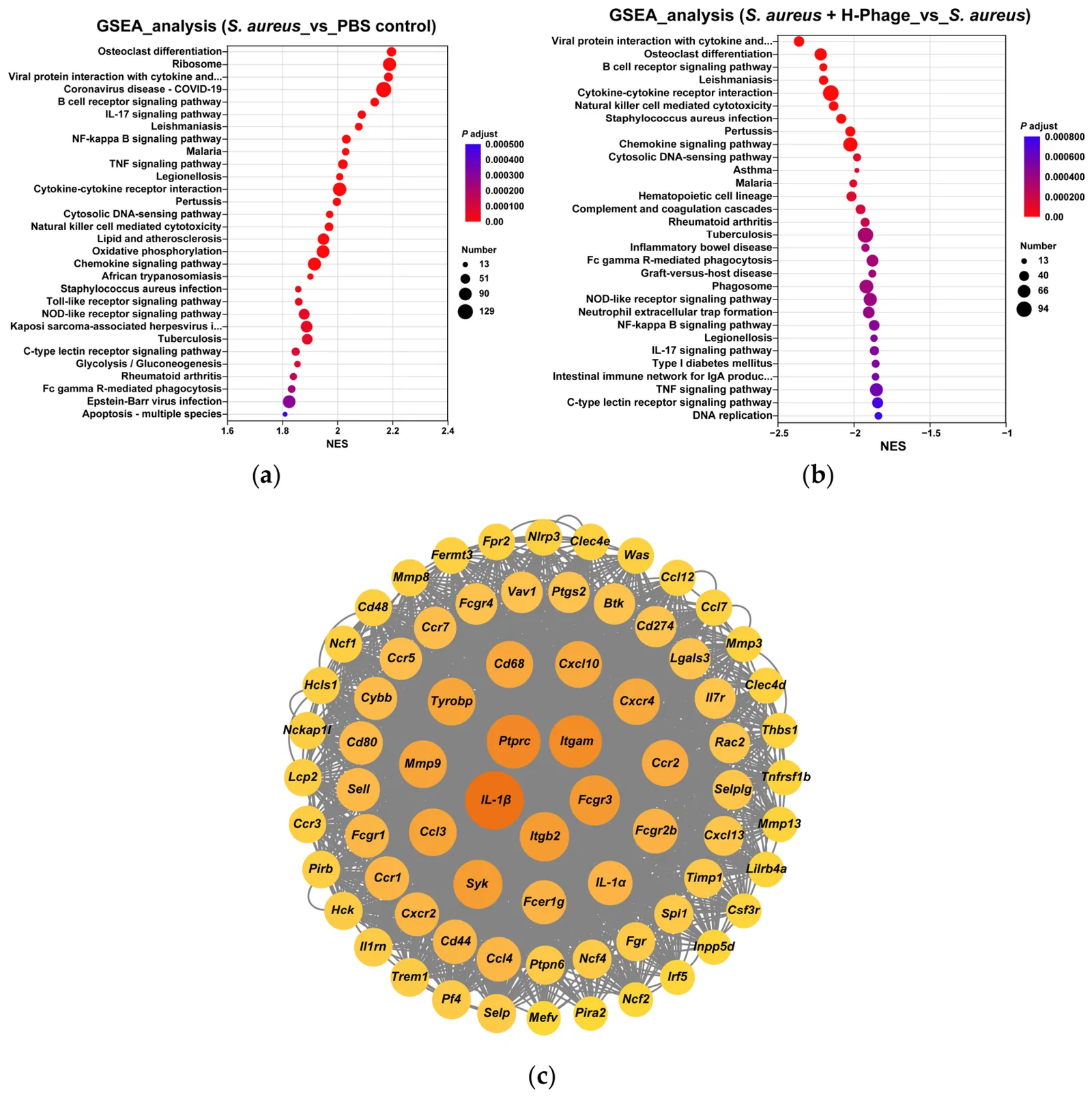
Gene set enrichment analysis (GSEA) and PPI network of overlapping DEGs between *S. aureus* vs. PBS control and *S. aureus* + H-Phage vs. *S. aureus* group. (**a**,**b**) GSEA of overlapping DEGs between *S. aureus* vs. PBS control and *S. aureus* + H-Phage vs. *S. aureus* group. (**c**) PPI network analysis of overlapping DEGs between *S. aureus* + H-Phage vs. *S. aureus* group.

To further determine whether specific signaling pathways were functionally activated or inhibited, Gene set enrichment analysis (GSEA) was performed across the KEGG database (Figure 5a,b). GSEA results revealed that the pro-inflammatory pathways were activated following *S. aureus* infection (positive NES). Conversely, high-dose phage intervention reversed these pathways, as evidenced by negative NES values. Specifically, phage therapy suppressed major pro-inflammatory signaling pathways, including cytokine–cytokine receptor interaction, the chemokine signaling pathway, *Staphylococcus aureus* infection, B cell receptor signaling, the TNF signaling pathway, NOD-like receptor signaling, and the NF-kappa B signaling pathway.

### 3.4. Identification of Core Regulatory Targets Through PPI Network Analysis

To identify the key molecular hubs underlying the therapeutic efficacy of phage therapy, a protein–protein interaction (PPI) network was constructed from the 448 reversed DEGs using the STRING database and visualized via Cytoscape. Based on network centrality metrics, prominent hub genes were identified, including *IL-1β*, *Ptprc*, *Itgam*, *Fcgr3*, *Itgb2*, *Syk*, *Ccl3*, *Mmp9*, and *Cxcl10* (Figure 5c). Functionally, these hub genes are critically associated with immune regulation and inflammatory response pathways, indicating that JXAU-SA-P1 mitigates mastitis by reducing this central inflammatory interaction.

### 3.5. RT-qPCR Validation of Hub Targets and Systemic Inflammatory Responses

**Figure 6:**
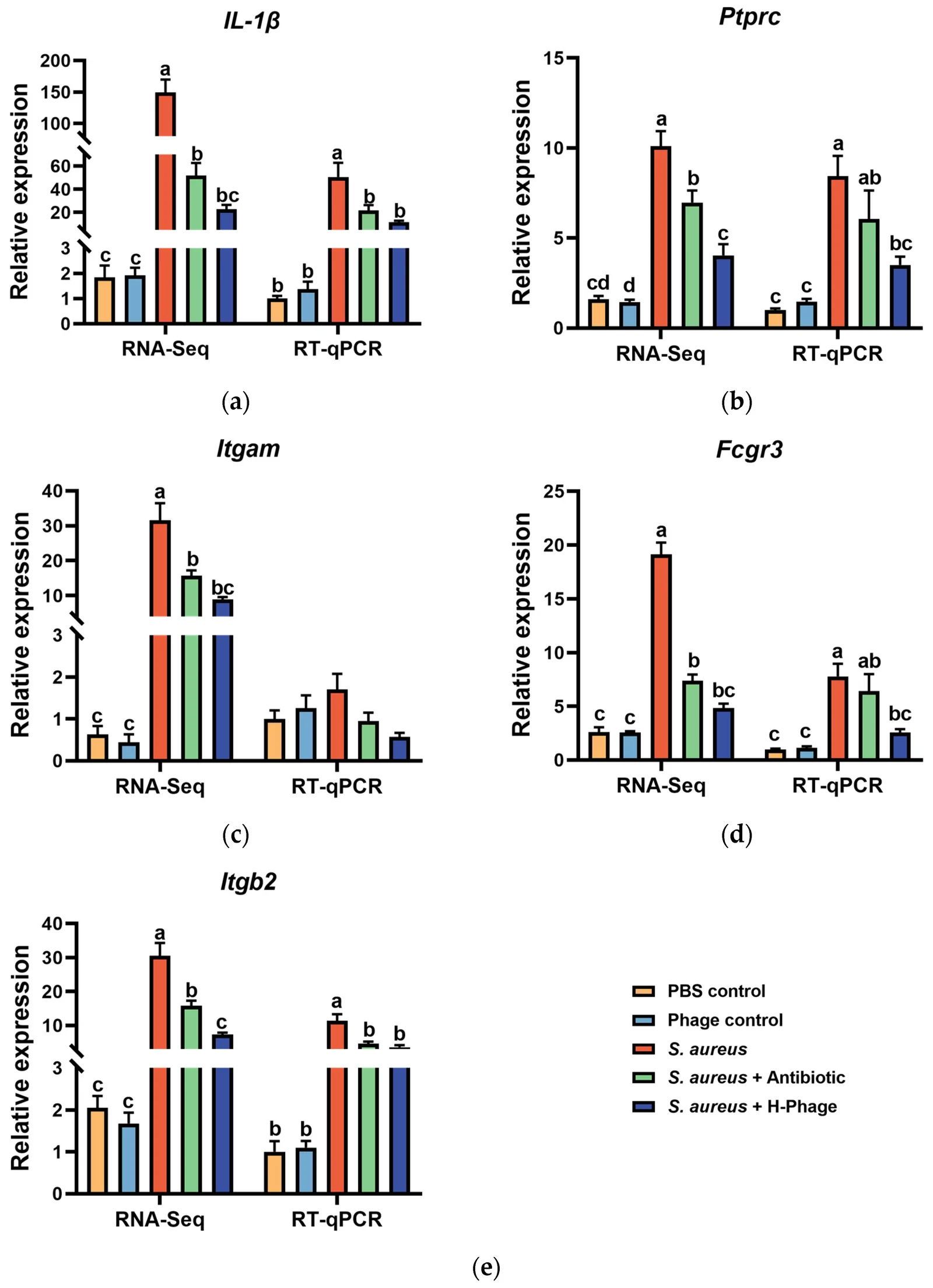
RT-qPCR validation (mean ± SEM, *n* = 6). (**a**–**e**) *IL-1β*, *Ptprc*, *Itgam*, *Fcgr3*, and *Itgb2* relative expression in RNA-seq and RT-qPCR. Different superscript letters indicate statistical differences among treatment groups (*p* < 0.05).

To validate the transcriptomic data, five hub genes (*IL-1β*, *Ptprc*, *Itgam*, *Fcgr3*, and *Itgb2*) prioritized from the PPI network were evaluated by RT-qPCR. Consistent with the RNA-seq profiles, four of the five genes (*IL-1β*, *Ptprc*, *Fcgr3*, and *Itgb2*) were significantly down-regulated in the *S. aureus* + H-Phage group relative to the *S. aureus* group (*p* < 0.05), while *Itgam* showed a non-significant trend of reduction in the RT-qPCR validation (Figure 6a–e). Furthermore, there was no significant difference in the expression of these genes between the PBS control and phage control groups (*p* > 0.05), validating both the RNA-seq accuracy and the biocompatibility of the phage.

**Figure 7:**
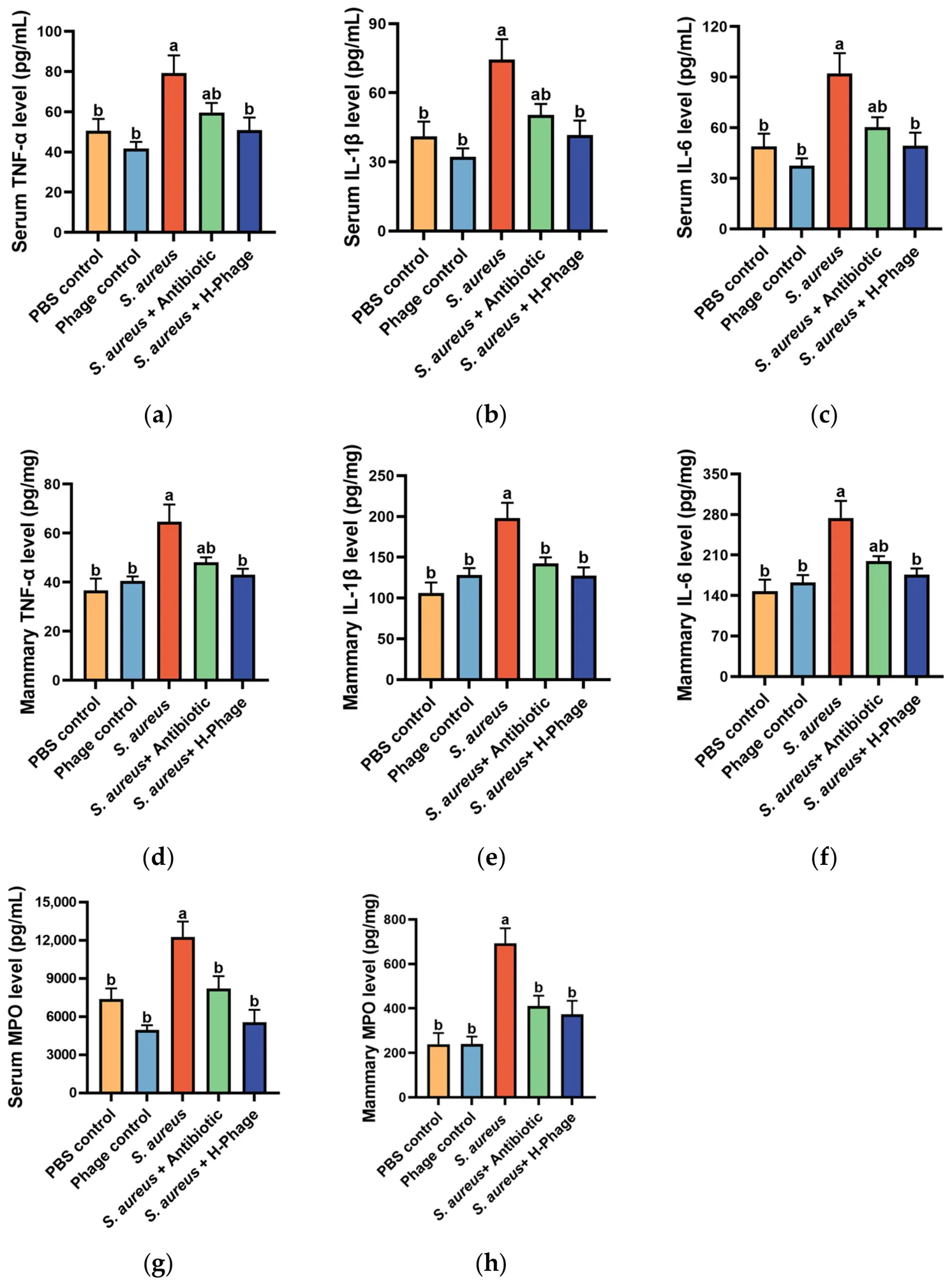
Pro-inflammatory cytokines and MPO levels in serum and mammary gland among the experimental groups (mean ± SEM; *n* = 6). (**a**–**c**) TNF-α, IL-1β and IL-6 levels in serum. (**d**–**f**) TNF-α, IL-1β and IL-6 levels in mammary gland. (**g**,**h**) MPO level in serum and mammary gland. Different superscript letters indicate statistical differences among treatment groups (*p* < 0.05).

Protein-level expression of key pro-inflammatory mediators was further characterized by ELISA (Figure 7). Paralleling the transcriptional down-regulation, high-dose phage treatment led to a significant decrease in TNF-α, IL-6, and IL-1β concentrations in both mammary glands and serum compared with the *S. aureus* group (*p* < 0.05). Additionally, myeloperoxidase (MPO) level in the serum and mammary tissues of the *S. aureus* + H-Phage group were significantly decreased (*p* < 0.05), confirming the attenuation of local neutrophil recruitment and activation. No significant variations in these inflammatory markers were observed between the PBS control and phage control group (*p* > 0.05).

**Figure 8:**
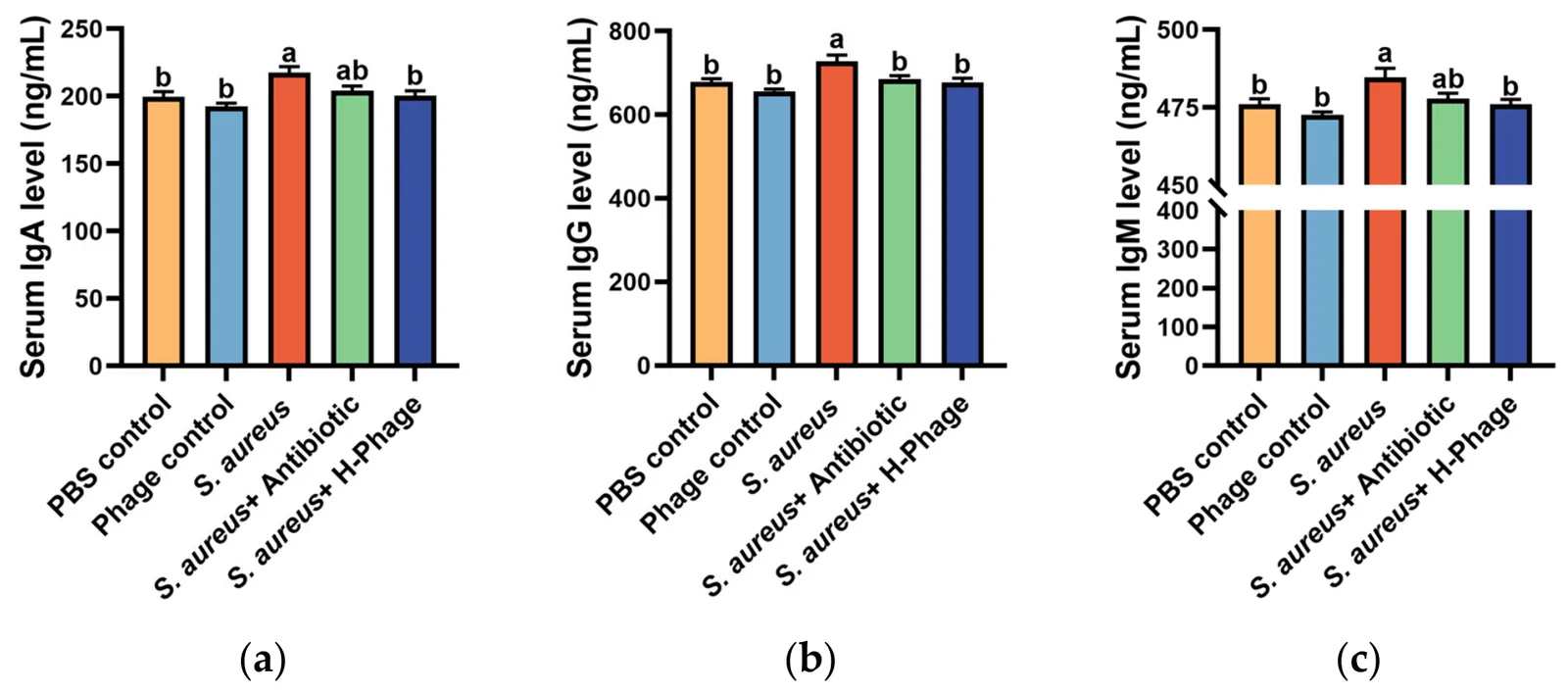
Immunoglobulins levels in serum among the experimental groups (mean ± SEM; *n* = 6). (**a**–**c**) IgA, IgG and IgM levels in serum. Different superscript letters indicate statistical differences among treatment groups (*p* < 0.05).

Finally, to determine whether phage intervention affected systemic immunity fluctuations, serum immunoglobulins were quantified (Figure 8). Serum levels of IgA, IgM, and IgG in the *S. aureus* + H-Phage group were significantly lower than those in the *S. aureus* group (*p* < 0.05), while no difference was observed between the PBS control and phage control group (*p* > 0.05). Collectively, these results verified that phage therapy effectively dampened pro-inflammatory cytokine secretion, limited neutrophil-mediated damage, and mitigated early systemic immune fluctuations.

## 4. Discussion

*S. aureus* is a common pathogen causing mastitis. It induces inflammation of the mammary gland tissue, leading to reduced milk production and causing huge economic losses in the dairy industry [26]. With the growing crisis of antimicrobial resistance and the potential risk of antibiotic residues in food, phages have emerged as a promising alternative to control bacterial infections due to their high host specificity, low susceptibility to resistance and absence of residues in vivo. In this study, utilizing a lactating mouse model of mastitis, the therapeutic efficacy and transcriptome regulatory mechanisms of the previously isolated phage JXAU-SA-P1 against mastitis caused by *S. aureus* were systematically assessed. Our findings showed that local intramammary administration of JXAU-SA-P1 significantly reduced *S. aureus* burden and alleviated histopathological damage induced by inflammation. In addition, the ideal biological control agent must have outstanding biocompatibility and biosafety properties. This study provides multi-level evidence for the safety of JXAU-SA-P1. Under identical dosing conditions, the phage control group had significantly lower phage titers than the *S. aureus* + H-Phage. This suggests that phage JXAU-SA-P1 maintains enhanced local persistence through host-dependent replication in the presence of *S. aureus*, sustaining a high local titer to promote bacterial reduction. Conversely, in the uninfected phage control group, viral particles were rapidly cleared from the mammary gland tissues [27]. Further analysis revealed that there was no difference in tissue inflammation and pathological damage between the phage control group and PBS control group [28,29]. Notably, transcriptome analysis revealed only eight DEGs between the phage control group and PBS control group. These findings indicate that JXAU-SA-P1 themselves do not trigger inflammatory responses in vivo.

*S. aureus* infection altered the transcriptional homeostasis of the mammary of the host [30]. We identified 2135 DEGs in the mammary glands between the PBS control group and the *S. aureus* group. These DEGs were mainly involved in immune system processes, signaling receptor activities, the TNF signaling pathway, and the chemokine signaling pathway. This indicates that the pathogen infection has activated the innate immunity and excessive inflammatory response of the mammary gland [31]. Notably, high-dose phage therapy reversed 448 infection response genes, which is a broader and more complex immunomodulatory profile than antibiotic treatment (which reversed 222 genes). KEGG enrichment analysis indicated that these 448 overlapping DEGs were significantly enriched in multiple immune and inflammation-related pathways. Meanwhile, GSEA results indicate that certain pathways activated following *S. aureus* infection were subsequently suppressed after JXAU-SA-P1 administration, including *S. aureus* infection, TNF signaling pathway, IL-17 signaling pathway, NF-kappa B signaling pathway and NOD-like receptor signaling pathway. It is hypothesized that JXAU-SA-P1 rapidly lyses *S. aureus*, thereby reducing the excessive activation of pathogen-associated molecular patterns. This secondary effect reduced the excessive activation of inflammatory cascade reactions and resolved the transcriptional storm [32,33]. Mammary epithelial cells and immune cells serve as the first line of defense against pathogen infection [34]. Patter recognition receptors (PRRs) on their cell membranes, such as Toll-like receptors (TLRs) and NOD-like receptors (NLRs), recognize pathogen-associated molecular patterns (PAMPs) from *S. aureus*, including lipoteichoic acid and peptidoglycan [35]. Following PRRs recognition, downstream NF-κB signaling pathways are activated, inducing the release of multiple pro-inflammatory cytokines, thereby intensifying the inflammatory response [36,37].

The protein–protein interaction (PPI) network identified several core regulatory hubs, including *IL-1β, Ptprc*, *Itgam*, and *Itgb2*, which were subsequently validated by RT-qPCR. *Itgam* and *Itgb2* encode the subunits of the Mac-1 integrin complex, which is a critical mediator of neutrophils’ adhesion and induces pro-inflammatory cytokine secretion [38]. The down-regulation of these molecules confirmed the intramammary administration of phage JXAU-SA-P1 can regulate the host innate response and alleviate the inflammation response. This was consisted with the abrogation of massive inflammatory cell infiltration observed in the histopathological sections and the significant reduction in myeloperoxidase (MPO) activity [39].

TNF-α, IL-6, and IL-1β, as key pro-inflammatory cytokines, exhibit expression levels that correlate positively with the severity of mouse mastitis and are frequently employed to assess the inflammatory status of mammary glands [31,40]. ELISA analyses confirmed that their expression was significantly down-regulated following JXAU-SA-P1 administration. For instance, phage CM8-1 was shown to significantly reduce mRNA expression of *TNF-α, IL-1β*, and *IL-6* in the *Klebsiella pneumoniae*-induced mastitis model [41]. Similarly, phage 4086-1 effectively lowered bacterial loads and levels of TNF-α and IL-6 in mammary tissue [24]. Furthermore, *S. aureus* infection may damage the blood–milk barrier of the mammary gland, allowing bacterial antigens to enter the blood stream and triggering a strong systemic immune response [42,43]. In the current study, the *S. aureus* group had higher serum IgA, IgG and IgM levels, indicating a stronger immune response to clearance the pathogen. In contrast, the high-dose phage treatment reversed this reaction, effectively attenuating the early systemic activation induced by pathogen. It should be noted that the absolute numerical changes in total serum immunoglobulins are relatively modest at 28 h post infection. However, these differences remain biologically relevant as early markers of blood–milk barrier disruption and systemic inflammatory effects. The ability of high-dose phage treatment to maintain these markers at an uninfected baseline underlines its effectiveness in rapidly clearing local pathogens, preserving tissue integrity and preventing systemic inflammation.

From a broader food safety perspective, the application of phage-based biocontrol agents in agricultural and veterinary settings is essential to ensure the safety and quality of meat, eggs and milk for human consumption. Yamik et al. demonstrated that *Escherichia coli* phages recovered from wastewater treatment stations effectively reduced bacterial counts and biofilm formation on contaminated meat [44]. Similarly, Yao et al. highlighted the dual use of sewage-derived salmonella phages in the treatment of chicken salmonella and the direct decontamination of food products [45]. Furthermore, Guan et al. utilized a sewage-isolated *Pseudomonas fluorescens* phage to express the endolysin LysPFX32, which exhibited antibacterial efficacy in the preservation of fresh retail pork meat [46]. Collectively, these studies highlight the potential of environmentally derived phages as a safe and antibiotic-free intervention to improve animal health and ensure food safety across the production chain.

## 5. Conclusions

Phage JXAU-SA-P1 significantly reduced *S. aureus* loads and alleviated histopathological damage of mammary glands induced by *S. aureus* infections. In addition, transcriptomic sequencing identified 448 overlapping DEGs between *S. aureus* and the *S. aureus +* H-Phage group. Gene set enrichment analysis revealed that phage administration suppressed major pro-inflammatory signaling pathways secondary to bacterial reduction. Furthermore, both RT-qPCR and ELISA validation confirmed that administration with phage JXAU-SA-P1 alleviated the systemic inflammation response and the innate immune activation by eliminating the primary inflammatory stimuli. More importantly, the safety of phage JXAU-SA-P1 has been demonstrated at the levels of inflammation, histopathology, and transcriptomics.

## Figures and Tables

**Table 1:** Primer sequences utilized for RT-qPCR analysis.

Gene	Accession Number	Primer Sequences (5′→3′)	Length (bp)
*IL-1β*	NM_008361.4	F: AGCTTCAAATCTCGCAGCAG R: TCTCCACAGCCACAATGAGT	189
*Ptprc*	NM_001420780.1	F: TTCTGGCCTTTGGATTTGCC R: CGTTGTGGTAGCATCACTGG	173
*Itgam*	NM_009915.2	F: AAGCACTTAGACCAGGCCAT R: TCACTCGATCTGCTGTCTCC	123
*Fcgr3*	NM_001405019.1	F: CCAGGTCCAATCCAGCTACA R: TTGTTCCTCCAGCTATGGCA	152
*Itgb2*	NR_157229.1	F: CTGCCACCTGGAGGATAACA R: CTCGGAAAGTTTGTGTGCCA	149
*β-actin*	NM_007393.5	F: TGGAATCCTGTGGCATCCAT R: GCTAGGAGCCAGAGCAGTAA	164

**Table 2:** Sequencing data statistics.

Sample	Raw Reads	Raw Bases	Clean Reads	Clean Bases	Q20 (%)	Q30 (%)	GC Content (%)
PBS control 1	37,914,356	5,725,067,756	37,737,606	5,673,150,066	99.40	96.98	49.22
PBS control 2	37,994,772	5,737,210,572	37,810,538	5,684,663,194	99.35	96.76	50.08
PBS control 3	59,107,818	8,925,280,518	58,801,714	8,847,143,777	99.33	96.69	50.41
PBS control 4	39,811,410	6,011,522,910	39,658,242	5,969,404,034	99.41	96.94	50.47
Phage control 1	40,614,104	6,132,729,704	40,418,510	6,079,757,612	99.37	96.80	49.62
Phage control 2	42,880,174	6,474,906,274	42,669,570	6,419,715,731	99.36	96.80	49.35
Phage control 3	39,354,644	5,942,551,244	39,168,074	5,890,245,419	99.37	96.84	49.60
Phage control 4	40,309,674	6,086,760,774	40,077,154	6,031,721,368	99.35	96.82	49.00
*S. aureus* 1	36,625,170	5,530,400,670	36,428,410	5,484,115,279	99.33	96.69	50.75
*S. aureus* 2	38,373,950	5,794,466,450	38,186,788	5,748,089,513	99.33	96.66	51.7
*S. aureus* 3	42,000,156	6,342,023,556	41,794,988	6,289,596,052	99.33	96.7	50.59
*S. aureus* 4	38,180,158	5,765,203,858	37,992,644	5,711,970,625	99.35	96.79	50.16
*S. aureus* + Antibiotic 1	61,565,764	9,296,430,364	61,229,790	9,206,810,904	99.33	96.74	50.32
*S. aureus* + Antibiotic 2	67,100,300	10,132,145,300	66,722,110	10,033,841,036	99.33	96.73	50.66
*S. aureus* + Antibiotic 3	40,682,556	6,143,065,956	40,483,654	6,093,499,993	99.35	96.75	51.07
*S. aureus* + Antibiotic 4	38,958,094	5,882,672,194	38,759,534	5,830,355,848	99.32	96.62	51.25
*S. aureus* + H-Phage 1	37,891,348	5,721,593,548	37,683,756	5,668,097,222	99.34	96.72	49.65
*S. aureus* + H-Phage 2	48,998,256	7,398,736,656	48,730,378	7,334,101,055	99.34	96.74	50.34
*S. aureus* + H-Phage 3	47,586,344	7,185,537,944	47,338,960	7,123,196,545	99.34	96.69	50.92
*S. aureus* + H-Phage 4	39,746,586	6,001,734,486	39,544,762	5,947,557,768	99.32	96.66	50.32

**Table 3:** Comparison of transcription data and reference genomes.

Sample	Total Reads	Total Mapped	Multiple Mapped	Unique Mapped
PBS control 1	37,737,606	36,823,526 (97.58%)	3,999,041 (10.60%)	32,824,485 (86.98%)
PBS control 2	37,810,538	36,956,233 (97.74%)	2,741,844 (7.25%)	34,214,389 (90.49%)
PBS control 3	58,801,714	57,566,456 (97.9%)	4,284,688 (7.29%)	53,281,768 (90.61%)
PBS control 4	39,658,242	38,797,736 (97.83%)	3,065,099 (7.73%)	35,732,637 (90.1%)
Phage control 1	40,418,510	39,481,290(97.68%)	3,682,566 (9.11%)	35,798,724 (88.57%)
Phage control 2	42,669,570	41,683,180 (97.69%)	3,719,147 (8.72%)	37,964,033 (88.97%)
Phage control 3	39,168,074	38,254,346 (97.67%)	2,842,373 (7.26%)	35,411,973 (90.41%)
Phage control 4	40,077,154	39,190,087 (97.79%)	3,527,194 (8.80%)	35,662,893 (88.99%)
*S. aureus* 1	36,428,410	35,559,125 (97.61%)	2,851,776 (7.83%)	32,707,349 (89.79%)
*S. aureus* 2	38,500,640	37,563,955 (97.57%)	2,643,139 (6.87%)	34,920,816 (90.70%)
*S. aureus* 3	41,721,428	40,763,037 (97.70%)	3,179,486 (7.62%)	37,583,551 (90.08%)
*S. aureus* 4	37,992,644	37,125,326 (97.72%)	3,142,371 (8.27%)	33,982,955 (89.45%)
*S. aureus* + Antibiotic 1	61,229,790	59,917,976 (97.86%)	4,479,448 (7.32%)	55,438,528 (90.54%)
*S. aureus* + Antibiotic 2	66,722,110	65,094,580 (97.56%)	5,357,693 (8.03%)	59,736,887 (89.53%)
*S. aureus* + Antibiotic 3	40,483,654	39,577,845 (97.76%)	3,090,794 (7.63%)	36,487,051 (90.13%)
*S. aureus* + Antibiotic 4	38,759,534	37,893,270 (97.77%)	2,825,994 (7.29%)	35,067,276 (90.47%)
*S. aureus* + H-Phage 1	37,683,756	36,807,509 (97.67%)	3,059,534 (8.12%)	33,747,975 (89.56%)
*S. aureus* + H-Phage 2	48,730,378	47,546,336 (97.57%)	3,778,516 (7.75%)	43,767,820 (89.82%)
*S. aureus* + H-Phage 3	47,338,960	46,224,196 (97.65%)	3,322,600 (7.02%)	42,901,596 (90.63%)
*S. aureus* + H-Phage 4	39,544,762	38,617,853 (97.66%)	3,026,464 (7.65%)	35,591,389 (90.0%)

## Data Availability

The original contributions presented in the study are included in the article; further inquiries can be directed to the corresponding authors. The complete genome sequence of bacteriophage JXAU-SA-P1 is available in the GenBank database under accession number PV172160.

## Abbreviations

The following abbreviations are used in this manuscript:

BP	Biological processes
CC	Cellular components
CFU	Colony-forming unit
DEGs	Differentially expressed genes
GSEA	Gene Set Enrichment Analysis
H&E	Hematoxylin and eosin
PBS	Phosphate-buffered saline
MF	Molecular function
MPO	Myeloperoxidase
NF-κB	Nuclear factor kappa B
PCA	Principal Component Analysis
PFU	Plaque-forming units
PPI	Protein–protein interaction

## References

[1] Varijakshapanicker P., Mckune S., Miller L., Hendrickx S., Balehegn M., Dahl G.E., Adesogan A.T. (2019). Sustainable Livestock Systems to Improve Human Health, Nutrition, and Economic Status. Anim. Front..

[2] Hogeveen H., Steeneveld W., Wolf C.A. (2019). Production Diseases Reduce the Efficiency of Dairy Production: A Review of the Results, Methods, and Approaches Regarding the Economics of Mastitis. Annu. Rev. Resour. Econ..

[3] Zhu S., Bian S., Lu J., Wang Y., Iqbal M., Kiani F.A., Dong H., Zhang X., Yuan Z., Liu F. (2026). Pathogenesis of Bovine Mastitis and Influence of the Gut Microbiota: A Review. J. Agric. Food Chem..

[4] Tomanić D., Samardžija M., Kovačević Z. (2023). Alternatives to Antimicrobial Treatment in Bovine Mastitis Therapy: A Review. Antibiotics.

[5] Song M., Tang Q., Ding Y., Tan P., Zhang Y., Wang T., Zhou C., Xu S., Lyu M., Bai Y. (2024). *Staphylococcus aureus* and Biofilms: Transmission, Threats, and Promising Strategies in Animal Husbandry. J. Anim. Sci. Biotechnol..

[6] Cheung G.Y.C., Bae J.S., Otto M. (2021). Pathogenicity and Virulence of *Staphylococcus aureus*. Virulence.

[7] Touaitia R., Ibrahim N.A., Touati A., Idres T. (2025). *Staphylococcus aureus* in Bovine Mastitis: A Narrative Review of Prevalence, Antimicrobial Resistance, and Advances in Detection Strategies. Antibiotics.

[8] Costa L.V., Gebara C., Zacaroni O.d.F., Freitas N.E., Silva A.N.d., Prado C.S., Nunes I.A., Cavicchioli V.Q., Duarte F.O.S., Lage M.E. (2024). Antibiotic Residues in Raw Cow’s Milk: A Systematic Review of the Last Decade. Foods.

[9] Gosselin V.B., Bodmer M., Schüpbach-Regula G., Steiner A., Meylan M. (2022). Survey on the Disposal of Waste Milk Containing Antimicrobial Residues on Swiss Dairy Farms. J. Dairy Sci..

[10] Navrátilova P., Borkovcova I., Stastkova Z., Bednarova I., Vorlova L. (2022). Effect of Cephalosporin Antibiotics on the Activity of Yoghurt Cultures. Foods.

[11] Vinayamohan P.G., Pellissery A.J., Venkitanarayanan K. (2022). Role of Horizontal Gene Transfer in the Dissemination of Antimicrobial Resistance in Food Animal Production. Curr. Opin. Food Sci..

[12] Strathdee S.A., Hatfull G.F., Mutalik V.K., Schooley R.T. (2023). Phage Therapy: From Biological Mechanisms to Future Directions. Cell.

[13] Chen J., Han J., Yang Z., Zhou W., He Y., Chen X., Li X., Zou T., You J. (2025). Bacteriophages as Potential Anti-Pathogenic Agents for Intestinal Health of Weaned Piglets in the Post-Antibiotic Era: An Updated Review. Animals.

[14] Alves D.R., Gaudion A., Bean J.E., Perez Esteban P., Arnot T.C., Harper D.R., Kot W., Hansen L.H., Enright M.C., Jenkins A.T.A. (2014). Combined Use of Bacteriophage K and a Novel Bacteriophage to Reduce *Staphylococcus aureus* Biofilm Formation. Appl. Environ. Microbiol..

[15] Yao X., Jia C., Qin T., Peng D., Huang L., Liu Y., Zhang S., Han Y., Yang Y., Li H. (2025). Characterization of vB_SaRS_FS19–1, a Newly Isolated Broad-Host-Range and Virulent Bacteriophage Infecting *Staphylococcus aureus* and Its Application in Foods. Food Chem..

[16] Liu Y., Ding M., Zou T., Chen X., Li X., Chen Y., Chen J., You J. (2026). Isolation and Characterization of a Lytic Bacteriophage against *Staphylococcus aureus*. BMC Vet. Res..

[17] Pelyuntha W., Wannasrichan W., Yamik D.Y., Khongkhai H., Yingkajorn M., Vongkamjan K. (2025). Exploring Two Polyvalent Lytic *Staphylococcus* Phages and Their Application in Controlling *Staphylococcus* spp. in Plant-Based Meat. Food Res. Int..

[18] Chen Y., Ding M., Chen X., Zou T., Liu Y., Chen J., You J. (2026). Strategically Isolated Bacteriophages Targeting ETEC K88 (F4) Alleviate Post-Weaning Diarrhea in Piglets via Modulation of Gut Microbiota and Inflammatory Responses. J. Anim. Sci. Biotechnol..

[19] Dong C., Chen Y., Ding M., Liu Y., Chen X., He Y., Zou T., Chen J., You J. (2024). Dietary Bacteriophage Administration Alleviates Enterotoxigenic Escherichia Coli-Induced Diarrhea and Intestinal Impairment through Regulating Intestinal Inflammation and Gut Microbiota in a Newly Weaned Mouse Model. Int. J. Mol. Sci..

[20] Guo M., Zhang Y., Wu L., Xiong Y., Xia L., Cheng Y., Ma J., Wang H., Sun J., Wang Z. (2024). Development and Mouse Model Evaluation of a New Phage Cocktail Intended as an Alternative to Antibiotics for Treatment of Staphylococcus Aureus-Induced Bovine Mastitis. J. Dairy Sci..

[21] Zhao C., Wu K., Hao H., Zhao Y., Bao L., Qiu M., He Y., He Z., Zhang N., Hu X. (2023). Gut Microbiota-Mediated Secondary Bile Acid Alleviates Staphylococcus Aureus-Induced Mastitis through the TGR5-cAMP-PKA-NF-κB/NLRP3 Pathways in Mice. npj Biofilms Microbiomes.

[22] Brouillette E., Malouin F. (2005). The Pathogenesis and Control of *Staphylococcus aureus*-Induced Mastitis: Study Models in the Mouse. Microbes Infect..

[23] Geng H., Zou W., Zhang M., Xu L., Liu F., Li X., Wang L., Xu Y. (2020). Evaluation of Phage Therapy in the Treatment of *Staphylococcus aureus*-Induced Mastitis in Mice. Folia Microbiol..

[24] Teng F., Xiong X., Zhang S., Li G., Wang R., Zhang L., Wang X., Zhou H., Li J., Li Y. (2022). Efficacy Assessment of Phage Therapy in Treating *Staphylococcus aureus*-Induced Mastitis in Mice. Viruses.

[25] Hu X., Guo J., Zhao C., Jiang P., Maimai T., Yanyi L., Cao Y., Fu Y., Zhang N. (2020). The Gut Microbiota Contributes to the Development of *Staphylococcus aureus*-Induced Mastitis in Mice. ISME J..

[26] Sharun K., Dhama K., Tiwari R., Gugjoo M.B., Iqbal Yatoo M., Patel S.K., Pathak M., Karthik K., Khurana S.K., Singh R. (2021). Advances in Therapeutic and Managemental Approaches of Bovine Mastitis: A Comprehensive Review. Vet. Q..

[27] Zeng X., Gao Y., Jiao S., Zhan Y., Cheng Y., Ma J., Wang H., Sun J., Wang Z., Yan Y. (2026). Phage ZH4 Rescues Murine Mastitis Infected with Hypervirulent Multidrug-Resistant Klebsiella Pneumoniae through Pathogen Elimination and Mammary Barrier Restoration. J. Dairy Sci..

[28] Cha K., Oh H.K., Jang J.Y., Jo Y., Kim W.K., Ha G.U., Ko K.S., Myung H. (2018). Characterization of Two Novel Bacteriophages Infecting Multidrug-Resistant (MDR) Acinetobacter Baumannii and Evaluation of Their Therapeutic Efficacy in Vivo. Front. Microbiol..

[29] Matsuzaki S., Yasuda M., Nishikawa H., Kuroda M., Ujihara T., Shuin T., Shen Y., Jin Z., Fujimoto S., Nasimuzzaman M.D. (2003). Experimental Protection of Mice against Lethal *Staphylococcus aureus* Infection by Novel Bacteriophage ϕMR11. J. Infect. Dis..

[30] Zhang H., Jiang H., Fan Y., Chen Z., Li M., Mao Y., Karrow N.A., Loor J.J., Moore S., Yang Z. (2018). Transcriptomics and iTRAQ-Proteomics Analyses of Bovine Mammary Tissue with Streptococcus Agalactiae-Induced Mastitis. J. Agric. Food Chem..

[31] Oviedo-Boyso J., Valdez-Alarcón J.J., Cajero-Juárez M., Ochoa-Zarzosa A., López-Meza J.E., Bravo-Patiño A., Baizabal-Aguirre V.M. (2007). Innate Immune Response of Bovine Mammary Gland to Pathogenic Bacteria Responsible for Mastitis. J. Infect..

[32] An S., Mi S., Chen S., Tang Y., Xing Y., Jing Y., Yu Y. (2025). Unraveling Key Transposable Elements in Pathogen-Induced Bovine Mastitis through Comparative in Vivo and in Vitro Transcriptomic Analysis. BMC Genom..

[33] Zhao Z., Hou Z., Chai J., Li C., Liu T., Li J., Zhang S., Zhang L., Ma Y. (2025). Mechanisms of Host-Bacterial Interactions during *Escherichia coli* or *Staphylococcus aureus* Infection of Mammary Epithelial Cells. Front. Vet. Sci..

[34] Bagnicka E., Szprynca A., Kościuczuk E., Jarczak J., Rzewuska M., Sakowski T., Zalewska M. (2026). The Effect of Staphylococcal Infection on Cathelicidin and β-Defensin mRNA Levels in Epithelial Cells Lining the Mammary Gland Cistern. Front. Immunol..

[35] Askarian F., Wagner T., Johannessen M., Nizet V. (2018). *Staphylococcus aureus* Modulation of Innate Immune Responses through Toll-like (TLR), (NOD)-like (NLR) and C-Type Lectin (CLR) Receptors. FEMS Microbiol. Rev..

[36] Akhtar M., Guo S., Guo Y., Zahoor A., Shaukat A., Chen Y., Umar T., Deng P.G., Guo M. (2020). Upregulated-Gene Expression of pro-Inflammatory Cytokines (TNF-α, IL-1β and IL-6) via TLRs Following NF-κB and MAPKs in Bovine Mastitis. Acta Trop..

[37] Khan M.Z., Khan A., Xiao J., Ma J., Ma Y., Chen T., Shao D., Cao Z. (2020). Overview of Research Development on the Role of NF-κB Signaling in Mastitis. Animals.

[38] Schneider P., Salamon H., Weizmann N., Nissim-Eliraz E., Lysnyansky I., Shpigel N.Y. (2023). Immune Profiling of Experimental Murine Mastitis Reveals Conserved Response to Mammary Pathogenic *Escherichia coli*, *Mycoplasma bovis*, and *Streptococcus uberis*. Front. Microbiol..

[39] Zhao L., Jin L., Yang B. (2023). Diosmetin Alleviates *S. Aureus*-Induced Mastitis by Inhibiting SIRT1/GPX4 Mediated Ferroptosis. Life Sci..

[40] Rainard P., Riollet C. (2006). Innate Immunity of the Bovine Mammary Gland. Vet. Res..

[41] Zhao W., Shi Y., Liu G., Yang J., Yi B., Liu Y., Kastelic J.P., Han B., Gao J. (2021). Bacteriophage Has Beneficial Effects in a Murine Model of *Klebsiella pneumoniae* Mastitis. J. Dairy Sci..

[42] Bao L., Sun H., Zhao Y., Feng L., Wu K., Shang S., Xu J., Shan R., Duan S., Qiu M. (2023). Hexadecanamide Alleviates *Staphylococcus aureus*-Induced Mastitis in Mice by Inhibiting Inflammatory Responses and Restoring Blood-Milk Barrier Integrity. PLoS Pathog..

[43] García V., Gómez M., Iglesias M., Sanjuan N., Gherardi M., Cerquetti M.C., Sordelli D. (1996). Intramammary Immunization with Live-Attenuated *Staphylococcus aureus*: Microbiological and Immunological Studies in a Mouse Mastitis Model. FEMS Immunol. Med. Microbiol..

[44] Yamik D.Y., Wannasrichan W., Khongkhai H., Watthanasakphuban N., Yingkajorn M., Pelyuntha W., Vongkamjan K. (2026). Revealing the Biological and Genomic Characteristics of *Escherichia Coli* Phages and Their Application in Reducing Biofilm Formation and Bacterial Counts in Contaminated Meat. Food Res. Int..

[45] Yao X., Luo S., Xie J., Jia C., Han Y., Yang Y., Zheng Y., Pan J., Geng J., Wang Y. (2026). Biological and Genomic Characteristics of a Polyvalent *Salmonella* Phage and Its Dual Application in Food Decontamination and Chick Salmonellosis Treatment. Food Res. Int..

[46] Guan P., Ming Z., Liu X., Shao Y., Pan H., Ding Y., Wang X. (2025). Expression and Characterization of a Novel Endolysin LysPFX32 as Potential Biological Antimicrobial Agent against *Pseudomonas fluorescens* for Pork Preservation. Int. J. Biol. Macromol..

